# Supplementary material for: Se-Enrichment Pattern, Composition, and Aroma Profile of Ripe Tomatoes after Sodium Selenate Foliar Spraying Performed at Different Plant Developmental Stages
Source: Plants (Basel). 2021 May 23;10(6):1050. doi: 10.3390/plants10061050 (PMC8224791; doi:10.3390/plants10061050)
Supplement: Supplementary file 1 [file plants-10-01050-s001.zip › plants-1239962-supplementary.pdf]

Supplementary Material

Table S1. VOCs identified in Red Ripe tomato samples by HS-SPME-GC-MS analysis. Retention time (RT), Retention Index (RI), quantifier ion (QI) and CAS number are reported.

Compound	RT (min)	RI	QI	CAS number
2-methylfuran	2.07	505	82	534-22-5
1-Penten-3-one	2.69	546	55	1629-58-9
1-Penten-3-ol	2.85	557	57	616-25-1
Pentanal	3.04	569	44	110-62-3
2-Pentenal, (E)-	4.45	653	83	1576-87-0
1-Pentanol	4.83	674	42	71- 41-0
3-Hexenal, (Z)-	5.58	709	69	6789-80-6
Hexanal	5.63	711	56	505-57-7
2,4-Dimethyl-1-	6.80	748	70	19549-87-2
2-Hexenal	7.32	765	83	6728-26-3
(Z)-3-Hexen-1-ol	7.42	768	67	928-96-1
1-Hexanol	7.88	783	56	111-27-3
Heptanal	8.96	914	70	111-71-7
2,4-hexadienal, (E,E)-	9.25	921	81	142-83-6
2-ethylfuran	9.41	925	81	3208-16-0
Methyl hexoate	9.77	934	74	106-70-7
4-Methylanisol	10.07	942	107	104-93-8
2-Heptenal, (E)-	11.02	966	83	18829-55-5
Methyl heptenone	12.07	992	43	110-93-0
2-pentylfuran	12.24	996	138	3777-69-3
β-Phellandrene	13.78	1038	93	555-10-2
Eucalyptol	13.87	1041	139	470-82-6
2-Isobutylthiazole	13.94	1043	99	18640-74-9
2-Octenal, (E)-	14.84	1068	70	2548-87-0
p-Tolualdehyde	15.79	1094	119	104-87-0
Guaiacol	15.86	1096	109	90-05-1
Linalool	16.39	1111	71	78-70-6
Nonanal	16.57	1116	57	124-19-6
Phenylethyl Alcohol	16.94	1126	91	60-12-8
Camphor	18.23	1162	95	76-22-2
Methyl salicylate	19.87	1208	120	119-36-8
Decanal	20.29	1220	70	112-31-2
Phenethyl Acetate	22.02	1269	104	103-45-7
Citral	22.51	1283	69	5392-40-5
Ethyl Decanoate	26.69	1409	88	110-38-3
2-Nonen-1-ol, (E)-	27.19	1425	82	31502-14-4
Geranylacetone	28.39	1464	69	3796-70-1
Dehydro-β-ionone	29.46	1499	177	17283-81-7
Pseudoionone	32.50	1600	69	3548-78-5
